# Serum ASGR2 level: an efficacy biomarker for balloon pulmonary angioplasty in patients with chronic thromboembolic pulmonary hypertension

**DOI:** 10.3389/fimmu.2024.1402250

**Published:** 2024-05-24

**Authors:** Wei-Jie Xu, Shang Wang, Qian-Hao Zhao, Jia-Yi Xu, Xiao-Yi Hu, Su-Gang Gong, Jing He, Hong-Ling Qiu, Ci-Jun Luo, Jian Xu, Hui-Ting Li, Ze-Pu Li, Lan Wang, Yu Shi, Ya-Lin Zhao, Rong Jiang

**Affiliations:** ^1^ Department of Clinical Laboratory, Shanghai Pulmonary Hospital, Tongji University School of Medicine, Shanghai, China; ^2^ Department of Cardiopulmonary Circulation, Shanghai Pulmonary Hospital, School of Medicine, Tongji University, Shanghai, China; ^3^ School of Life Science and Technology, Tongji University, Shanghai, China; ^4^ Department of Cardiology, Affiliated Renhe Hospital of Shanghai University, Shanghai, China; ^5^ Department of Cardiology, Yantai Yu-Huangding Hospital, Medical College of Qingdao University, Yantai, China; ^6^ Department of Respiratory Critical Care Medicine, The First Hospital of Kunming, Kunming, China

**Keywords:** chronic thromboembolic pulmonary hypertension, balloon pulmonary angioplasty, proteomics, asialoglycoprotein receptor 2, immune

## Abstract

**Background:**

This study aimed to employ plasma proteomics to investigate the molecular changes, pathway alterations, and potential novel biochemical markers associated with balloon pulmonary angioplasty (BPA) in patients with chronic thromboembolic pulmonary hypertension (CTEPH).

**Methods:**

Pre- and post-BPA plasma samples from five CTEPH patients in the PRACTICE study were analyzed to identify differentially expressed proteins. Proteomic and bioinformatics analyses were conducted, and the identified proteins were further validated using ELISA assays in a separate cohort of the same study. Correlation and multivariate regression analyses were performed to investigate the associations between these differentially expressed proteins and clinical parameters.

**Results:**

Significantly higher serum levels of asialoglycoprotein receptor 2 (ASGR2) were detected in 5 CTEPH patients compared to those in healthy individuals but decreased significantly after successful BPA procedures. The decrease in serum levels of ASGR2 after the completion of BPA procedures was further validated in a separate cohort of 48 patients with CTEPH [0.70 (0.51, 1.11) ng/mL vs. 0.38 (0.27, 0.59) ng/mL, *P* < 0.001]. Significant associations were found between the pre-BPA ASGR2 level and clinical parameters, including neutrophil percentage (R = 0.285, *P* < 0.05), platelet (PLT) count (R = 0.386, *P* < 0.05), and high-density lipoprotein cholesterol (HDL-C) before BPA (R = -0.285, *P* < 0.05). Significant associations were detected between post-BPA serum ASGR2 levels and lymphocyte percentage (LYM%) (R = 0.306, *P* < 0.05), neutrophil-to-lymphocyte ratio (R = -0.294, *P* < 0.05), and pulmonary vascular resistance after BPA (R = -0.35, *P* < 0.05). Multivariate stepwise regression analysis revealed that pre-BPA ASGR2 levels were associated with HDL-C and PLT count (both *P* < 0.001), while post-BPA ASGR2 levels were associated with LYM% (*P* < 0.05).

**Conclusion:**

Serum levels of ASGR2 may be a biomarker for the effectiveness of BPA treatment in CTEPH patients. The pre-BPA serum level of ASGR2 in CTEPH patients was associated with HDL-C and the PLT count. The post-BPA serum level of ASGR2 was correlated with the LYM%, which may reflect aspects of immune and inflammatory status.

## 1 Introduction

Chronic thromboembolic pulmonary hypertension (CTEPH) is a pulmonary vascular disease characterized by pulmonary artery thrombus, pulmonary vascular remodeling leading to vascular stenosis or occlusion, and progressive elevated pulmonary artery pressure, ultimately leading to right heart failure (1). In addition to thrombotic factors, immune function and inflammatory status contribute significantly to the development of CTEPH, which is characterized by increased activation of innate and adaptive immune cells that promote inflammation and vascular disease (2).

In addition to riociguat, a targeted medicine for CTEPH, and pulmonary endarterectomy, balloon pulmonary angioplasty (BPA) is an appropriate alternative therapeutic option for patients who are not eligible for surgery or who experience persistent or recurrent pulmonary hypertension after PEA. Balloon pulmonary angioplasty effectively improves hemodynamics, right ventricular (RV) function, exercise capacity, symptoms, and prognosis (3, 4). Moreover, BPA treatment has been demonstrated to decrease interleukin (IL)-6 and C-reactive protein levels in CTEPH patients, indicating its potential to improve systemic inflammation (5). Although transcriptome sequencing and bioinformatics analysis have been used to investigate the pathogenesis of CTEPH (6), proteomic studies assessing the therapeutic efficacy of BPA are lacking.

In this study, we conducted proteomic profiling to identify changes in protein expression between pre-BPA and post-BPA samples from patients with CTEPH. Differentially expressed proteins were identified, and their correlations with clinical parameters were investigated in a cohort of patients enrolled in the PRACTICE study. We aimed to utilize plasma proteomics to uncover molecular changes, pathway alterations, and potential novel biochemical markers associated with BPA treatment in CTEPH patients.

## 2 Materials and methods

### 2.1 Study population

The CTEPH patients included in our study were selected from the PRACTICE study (ChiCTR2000032403) (7). The PRACTICE study, which was a prospective, randomized controlled study conducted at a single center, aimed to compare the effectiveness and safety of combining BPA with riociguat versus riociguat monotherapy in patients with inoperable CTEPH. All patients enrolled in the study met the diagnostic criteria for CTEPH in the ESC/ERS 2022 guidelines (8). These criteria included a mean pulmonary artery pressure (mPAP) > 20 mmHg, pulmonary artery wedge pressure (PAWP) ≤ 15 mmHg, and pulmonary vascular resistance (PVR) > 2 Wood units (WUs) via right heart catheterization (RHC). In the BPA group, patients initially underwent pre-RHC and received riociguat therapy for management of pulmonary arterial hypertension (PAH). Following a 3-month stabilization period, hospitalizations were scheduled at monthly intervals for BPA sessions until sufficient improvement in pulmonary vasculature patency was achieved. A post-RHC assessment was conducted three days after the final BPA procedure to evaluate hemodynamics. The study protocol was conducted in accordance with the revised Declaration of Helsinki, and approval was obtained from the Ethics Committee of the Shanghai Pulmonary Hospital (L20-385-1). Written informed consent was obtained from all participants.

Pre-BPA and post-BPA assessments included the World Health Organization functional class (WHC FC), 6-minute walk distance (6MWD), routine blood tests, lipid profile, N-terminal pro-B-type natriuretic peptide (NT-proBNP) levels, RHC and echocardiography parameters. Parameters that were measured and recorded during the RHC included mPAP, PAWP, cardiac output (CO), and PVR (9). Echocardiographic parameters included right atrial area (RAA), right ventricular end-diastolic transverse diameter (RVEDTD), right ventricular end-diastolic longitudinal diameter, right atrial transverse diameter (RATD), right atrial longitudinal diameter (RALD), and tricuspid annular plane systolic excursion (TAPSE) (10).

Pre- and post-BPA serum samples from five CTEPH patients in the PRACTICE study were analyzed to identify differentially expressed proteins. Proteomic and bioinformatics analyses were conducted, and the identified proteins were further validated using enzyme-linked immunosorbent assay (ELISA) in a separate cohort of 48 CTEPH patients in the PRACTICE study.

### 2.2 Sample collection

Blood samples were centrifuged at 3000×g for 10 minutes at 4°C to obtain serum, which was stored at -80°C for ELISA testing.

### 2.3 Mass spectrometry

Plasma samples from five patients were subjected to analysis, which involved protein extraction, peptide digestion, chromatographic fractionation, and liquid chromatography-tandem mass spectrometry data acquisition.

### 2.4 Bioinformatics analysis

The analysis consisted of three stages: quantitative, differential expression and functional analysis (11).

#### 2.4.1 Quantitative analysis

The detected proteins were compared with contents of the Swiss-Prot human protein database, and the number and overlap of relationships between groups were counted.

#### 2.4.2 Differential expression analysis

The quantifiable proteins identified through mass spectrometry were carefully selected, and multiple rounds of comprehensive protein quantification experiments were conducted. The ratio of the mean values before and after treatment was calculated to assess differential expression, while the P value was used to determine the significance of protein level comparisons between the two groups.

#### 2.4.3 Functional analysis

For subcellular localization and domain analysis, CELLO and InterProScan software, respectively, were used to investigate the functional regions and biological roles of proteins (12). Blast2GO software annotates differentially expressed protein sets with the Gene Ontology (GO) database, categorizing them into biological processes, molecular functions, and cellular components (13). KASS interprets and annotates proteins based on the KEGG pathway database (13). Fisher’s exact test was performed to compare the distribution of gene ontology GO classifications and KEGG pathways in the target protein set with that in the overall protein set, enabling enrichment analysis.

### 2.5 Enzyme-linked immunosorbent assay

The plasma concentrations of apolipoprotein C1 (APOC1), asialoglycoprotein receptor 2 (ASGR2) and heparan sulfate proteoglycan 2 (HSPG2) were selected from 13 differentially expressed proteins and measured using a Human APOC1 ELISA Kit (catalog# EH0529, FineTest^®^), a Human ASGR2 ELISA Kit (catalog#EH2669, FineTest^®^) and a Human HSPG2 ELISA Kit (catalog#EH0955, FineTest^®^), respectively. The detection limits were as follows: APOC1, 9.375−600 ng/mL, ASGR2 0.156−10 ng/mL, and HSPG2 0.625−40 ng/mL. The protein concentrations were determined by measuring the absorbance at 450 nm and then using a standard curve for calculations.

### 2.6 Data analysis

In the proteomics analysis, logarithmic transformation with a base of 2 was applied to normalize the data. The Student’s t test was then used to calculate *P* values for statistical analysis. Due to the limited number of significant differences, genes exhibiting an expression fold change ≥ 1.18 were considered significantly upregulated, while genes with an expression fold change < 0.85 were considered significantly downregulated, with a significance level of *P* < 0.05.

The serum ASGR2 levels and clinical parameters with a normal distribution are presented as the means ± standard deviations. Nonnormally distributed data are reported as medians (interquartile ranges). The Mann-Whitney U test was used to compare the serum concentrations of differentially expressed proteins (DEPs) between the CTEPH patients and individuals. The pre- and post-BPA changes in DEPs and clinical parameters were assessed using either the Wilcoxon signed-rank test or paired t test, depending on whether the data were normally distributed.

Spearman correlation analysis was performed to explore the relationships between serum ASGR2 levels and clinical parameters before and after BPA treatment.

Multiple linear stepwise regression analyses were conducted to investigate the associations between ASGR2 and clinical parameters. To address nonnormally distributed data, the natural logarithm (ln) was utilized to transform the values into normally distributed data. Covariance tests were performed on the relevant variables. GraphPad Prism 9 and IBM SPSS 23.0 software were used. A *P* value less than 0.05 was considered statistically significant.

## 3 Results

### 3.1 Characteristics of CTEPH patients undergoing proteomic analysis

The average age of the five patients was 59.2 ± 8.7 years, and three of them were female. After the completion of BPA treatment, the mPAP significantly decreased from 40.6 ± 6.9 mmHg to 19.4 ± 4.6 mmHg (*P* < 0.01). Additionally, the PVR decreased significantly from 9.6 ± 4.2 mmHg to 3.1 ± 0.4 mmHg (*P* = 0.023) (**Table 1**).

**Table 1:** Clinical information of 5 CTEPH patients undergoing proteomic analysis.

Patient	Gender	Age (years)	6MWD (m)	NT-proBNP (pg/ml)	RAP (mm Hg)	mPAP (mm Hg)	PAWP (mm Hg)	CO (L/min)	PVR (Wood U)	S_V_O_2_ (%)
Pre-BPA										
1	Female	65	465	151.5	1	40	6	2.41	14.10	59.8
2	Male	50	375	126.0	1.0	45	5	2.97	13.47	60.5
3	Female	57	420	235.0	3.0	31	8	6.07	3.79	70.5
4	Female	53	520	60.0	0.0	49	1	5.57	8.62	48.7
5	Male	71	195	4281.0	1	38	10	3.40	8.24	56.6
Post-BPA										
1	Female	65	450	136.5	1	14	2	3.9	3.05	69.9
2	Male	50	390	29.0	1	15	4	3.20	3.44	60.7
3	Female	57	400	125.0	1	21	6	4.23	3.55	70.3
4	Female	53	520	65.0	1	24	9	5.90	2.54	69.5
5	Male	71	455	353.7	0	23	6	5.73	2.97	58.3

6MWD, 6-minute walking distance; NT-proBNP, N-terminal pro B-type natriuretic peptide; RAP, right atrial pressure; mPAP, mean pulmonary artery pressure; PAWP, pulmonary artery wedge pressure; CO, cardiac output; PVR, pulmonary vascular resistance; S_V_O_2_, saturation of mixed venous blood oxygen.

### 3.2 Proteomic and bioinformatics analysis

A total of 625 proteins were identified in the quantitative analysis, with 559 proteins overlapping between the groups. Furthermore, 21 proteins were exclusively detected in the pre-BPA group, while 45 proteins were exclusively detected in the post-BPA group.

**Figure 1:**
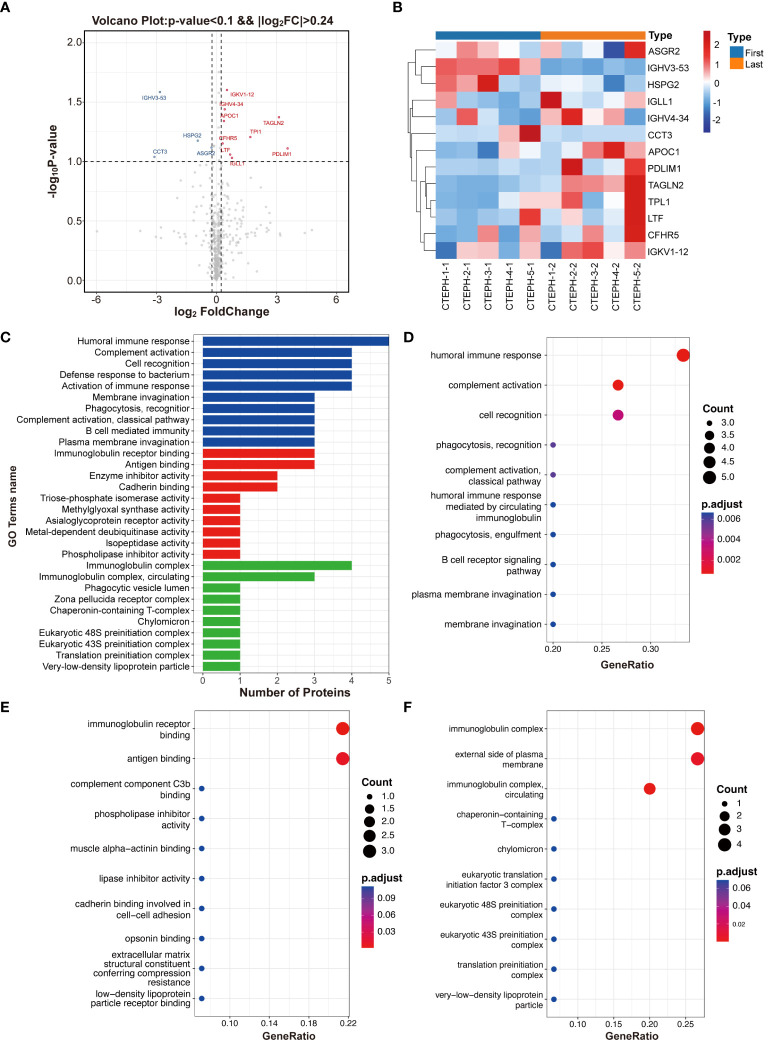
Differentially expressed protein identification and functional enrichment analysis. **(A)** Volcano plot. **(B)** Heatmap of differentially expressed proteins. **(C)** Bar plot showing the number of differentially expressed proteins at the secondary functional annotation level of GO. **(D)** The top 10 enriched terms in the biological process category. **(E)** The top 10 enriched terms in the molecular function category. **(F)** The top 10 enriched terms in the cellular component category.

Volcano plots were generated based on fold change values ≥ 1.18 or ≤ 0.85 and *P* values < 0.1 (**Figure 1A**). Four downregulated differentially expressed proteins encoded by the genes IGHV3-53, CCT3, HSPG2, and ASGR2 were identified. Nine upregulated differentially expressed proteins encoded by the genes TAGLN2, PDLIM1, TPI1, APOC1, CFHR5, LTF, IGLL1, IGHV4-34, and IGKV1-12 were also identified (**Figure 1B**).

The GO functional annotation revealed significant enrichment of differentially expressed proteins in key biological processes (such as humoral immune response, complement activation, and cell recognition), emphasizing their relevance to immune function and cell interactions (**Figures 1C, D**). In terms of molecular function, these proteins were primarily involved in immunoglobulin receptor binding and antigen binding (**Figure 1E**) and were also significantly enriched in cellular components, such as immunoglobulin complexes and the external side of the plasma membrane (**Figure 1F**). Notably, APOC1, HSPG2, and ASGR2-encoded proteins were frequently found in these enriched pathways, suggesting their potential as characteristic differentially expressed proteins for further validation.

According to the KEGG pathway analysis, the top pathways were phototransduction, fructose and mannose metabolism, primary immunodeficiency, cholesterol metabolism, glycolysis/gluconeogenesis, long-term potentiation, renin secretion, amphetamine addiction, inositol phosphate metabolism and amino acid biosynthesis.

### 3.3 Detection of DEPs in serum samples from 5 CTEPH patients

**Figure 2:**
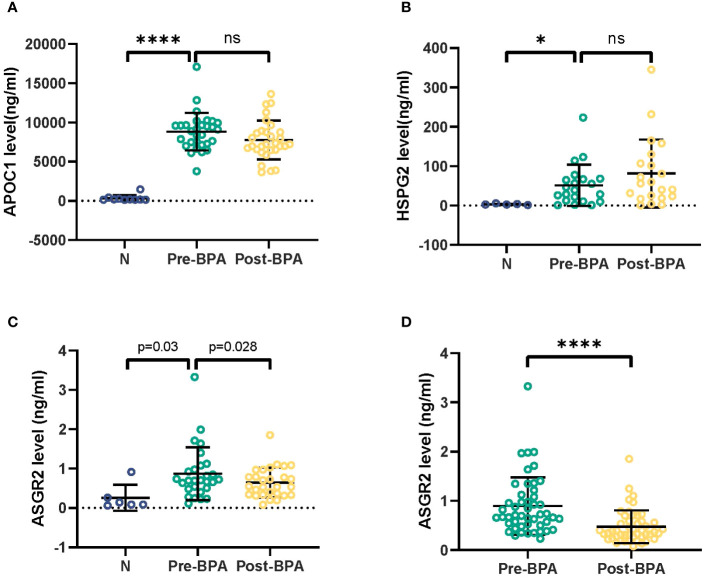
Detection of APOC1, HSPG2 and ASGR2 in serum samples from 5 CTEPH patients. CTEPH, Chronic thromboembolic pulmonary hypertension. *p< 0.05; ns, not significant; ****p<0.001.

The serum levels of APOC1 and HSPG2 in 5 CTEPH patients were significantly greater than those in healthy individuals. However, no significant differences were observed between the pre- and post-BPA treatments. Compared with those in healthy individuals, significantly greater serum levels of ASGR2 were detected in 5 CTEPH patients (*P* = 0.03) but decreased significantly after successful BPA procedures (*P* = 0.028) (**Figure 2**).

### 3.4 Validation of ASGR2 in the PRACTICE cohort

Following the completion of BPA treatment, a notable decrease in the serum ASGR2 concentration was observed in the 48 CTEPH patients in the PRACTICE study [0.70 (0.51, 1.11) ng/mL vs. 0.38 (0.27, 0.59) ng/mL, *P* < 0.001]. Of the patients, 24 were female, with a mean age of 61.8 ± 11.3 years.

Following successful BPA therapy, a significant improvement was observed in the WHO FC (*P* < 0.001). Hemodynamically, post-BPA treatment substantially reduced the mPAP and PVR (45.7 ± 11.1 mmHg vs. 25.7 ± 6.3 mmHg and 9.3 ± 6.0 mmHg vs. 3.3 ± 0.8 mmHg, respectively; both *P* < 0.001). Additionally, CO and SvO_2_ significantly increased [4.8 ± 1.2 L/min vs. 5.6 ± 1.1 L/min, *P* = 0.004; 59.5 ± 8.8% vs. 68.9 ± 6.8%, *P* < 0.001, respectively]. Through echocardiography, significant reductions were observed in RV structure parameters, including RAA, RATD, RALD, and RVEDTD [18.50 (15.9, 23.2) mm² vs. 14.1 (11.5, 17.8) mm², *P* < 0.01; 18.5 (15.9, 23.2) cm vs. 14.1 (11.5, 17.8) cm, *P* < 0.001; 5.2 ± 0.8 cm vs. 4.6 ± 0.8 cm, *P* < 0.001; 4.0 ± 0.7 cm vs. 3.5 ± 0.5 cm, *P* < 0.001, respectively]. A significant increase in RV systolic function was observed for the TAPSE [11.0 (9.2, 12.0) mm vs. 13.0 (11.0, 14.0) mm, *P* < 0.001] (**Table 2**).

**Table 2:** Hemodynamic and echocardiographic changes pre- and post-BPA.

Variable	Pre-BPA n = 48	Post-BPA n = 48	*P* value
Age (y)	61.8 ± 11.3		
Female, n (%)	24 (50)		
ASGR2, ng/mL	0.70 (0.51, 1.11)	0.38 (0.27,0.59)	< 0.001
WHO FC, n (%)			< 0.001
II	5 (10.4)	37 (77.1)	
III	42 (87.5)	11 (22.9)	
IV	1 (2.1)	0 (0)	
6MWD, m	356.5 ± 104.1	442.3 ± 67.8	< 0.001
Hemodynamics			
mPAP, mm Hg	45.7 ± 11.1	25.7 ± 6.3	< 0.001
PAWP, mm Hg	8.0 ± 3.4	7.8 ± 3.5	0.736
CO, L/min	4.8 ± 1.2	5.6 ± 1.1	0.004
PVR, Wood U	9.3 ± 6.0	3.3 ± 0.8	< 0.001
S_V_O_2_, %	59.5 ± 8.8	68.9 ± 6.8	< 0.001
Echocardiography			
RAA, mm^2^	18.5 (15.9, 23.2)	14.1 (11.5, 17.8)	< 0.001
RATD, cm	4.4 ± 0.9	3.6 ± 0.6	< 0.001
RALD, cm	5.2 ± 0.8	4.6 ± 0.8	< 0.001
RVEDTD, cm	4.0 ± 0.7	3.5 ± 0.5	< 0.001
RVEDLD, cm	6.5 (5.8, 7.0)	6.6 (6.0, 7.0)	0.166
sPAP, mmHg	71.5 (52.3, 97.8)	42.0 (37.3, 49.0)	< 0.001
TAPSE, mm	11.0 (9.2, 12.0)	13.0 (11.0, 14.0)	< 0.001

Values are expressed as the mean ± SD or median (quartile range). BPA, balloon pulmonary angioplasty; ASGR2, asialoglycoprotein receptor 2; WHO FC, World Health Organization functional class; 6MWD, 6-minute walking distance; mPAP, mean pulmonary artery pressure; PAWP, pulmonary artery wedge pressure; CO, cardiac output; PVR, pulmonary vascular resistance; S_V_O_2_, saturation of mixed venous blood oxygen; RAA, right atrial area; RATD, right atrial transverse dimension; RALD, right atrial longitudinal dimension; RVEDTD, right ventricular transverse dimension; RVEDLD, right ventricular longitudinal dimension; sPAP, systolic pulmonary artery pressure; TAPSE, tricuspid annular plane systolic excursion.

The levels of NT-proBNP decreased significantly [596.3 (110.4−1763.5) pg/ml to 125.5 (54.8−178.6) pg/ml, *P* < 0.001]. The white blood cell counts increased [5.7 (4.7−6.8) × 10^9^/L vs. 6.6 (4.9−8.2) × 10^9^/L) (*P* = 0.011), accompanied by an increase in the neutrophil percentage (NEU%) [56.0 ± 9.30% vs. 67.0 ± 10.9%, *P* = 0.041]. However, there was a decrease in the lymphocyte percentage (LYM%) [32.7 ± 8.1% vs. 23.5 ± 9.2%, *P* = 0.005] and eosinophil percentage [2.8 ± 2.9% vs. 1.9 ± 2.9%, *P* = 0.0013]. These changes resulted in an elevated neutrophil-to-lymphocyte ratio (NLR) [1.9 ± 0.8 vs. 3.4 ± 1.6, *P* = 0.021]. Platelet counts remained unchanged.

In terms of lipid metabolism, a significant increase in apolipoprotein A-1 (ApoA-I) levels was observed [1.3 ± 0.3 g/L vs. 1.4 ± 0.2 g/L, *P* = 0.003). No significant changes were detected in total cholesterol, triglyceride, high-density lipoprotein cholesterol (HDL-C), low-density lipoprotein cholesterol (LDL-C), small dense LDL cholesterol (SDLDL-C), ApoB, ApoE, or lipoprotein (a) levels (**Table 3**).

**Table 3:** Blood test changes between pre- and post-BPA.

Variable	Pre-BPA n = 48	Post-BPA n = 48	*P* value
NT-proBNP, pg/ml	596.3 (110.4, 1763.5)	125.5 (54.8, 178.6)	< 0.001
ESR, mm/h	16.0 ± 12.0	14.3 ± 11.5	0.522
hs-CRP, mg/L	5.6 ± 8.9	4.1 ± 6.0	0.327
Blood routine			
WBC, ×10^9^/L	5.7 (4.7, 6.8)	6.6 (4.9, 8.2)	0.011
NEU%, %	56.0 ± 9.3	67.0 ± 10.9	0.041
LYM%, %	31.7 (26.2, 39.5)	20.9 (16.2, 30.4)	0.005
NLR	1.9 ± 0.8	3.4 ± 1.6	0.021
EO%, %	2.8 ± 2.9	1.9 ± 2.9	0.013
PLT, ×10^9^/L	225.5 ± 85.4	224.3 ± 77.6	0.839
Renal function			
UA, μmol/L	355.0 (294.5, 445.3)	339.5 (278.6, 400.3)	0.070
BUN, mmol/L	6.1 ± 1.9	6.2 ± 1.5	0.666
Crea, μmol/L	68.6 ± 16.0	66.5 ± 14.4	0.167
Lipid metabolism			
TCH, mmol/L	4.2 ± 0.8	4.5 ± 0.9	0.115
TG, mmol/L	1.4 ± 0.6	1.4 ± 0.7	0.881
HDL-C, mmol/L	1.1 ± 0.3	1.1 ± 0.3	0.213
LDL-C, mmol/L	2.7 ± 0.7	2.9 ± 0.6	0.180
SDLDL-C, mmol/L	0.8 ± 0.3	0.7 ± 0.3	0.217
ApoA-I, g/L	1.3 ± 0.3	1.4 ± 0.2	0.003
ApoB, g/L	2.0 ± 8.3	0.9 ± 0.2	0.192
ApoE, g/L	36.4 (29.4, 45.0)	36.5 (28.9, 48.0)	0.765
LP (a), mg/L	124.9 (79.4, 281.5)	143.8 (92.6, 301.1)	0.549

Values are expressed as the mean ± SD or median (quartile range). BPA, balloon pulmonary angioplasty; NTproBNP, N-terminal pro B-type natriuretic peptide; ESR, erythrocyte sedimentation rate; hs-CRP, high-sensitivity C-reactive protein; WBC, white blood cell; NEU%, neutrophil percentage; LYM%, lymphocyte percentage; NLR, neutrophil to lymphocyte ratio; EO%, eosinophil percentage; PLT, platelet; UA, uric acid; BUN, blood urea nitrogen; Crea, creatinine; TCH, total cholesterol; TG, triglyceride; HDL-C, high density lipoprotein cholesterol; LDL-C, low density lipoprotein cholesterol; SDLDL-C, small dense low density lipoprotein cholesterol; ApoA, Apolipoprotein A; ApoB, Apolipoprotein B; ApoE, Apolipoprotein E; LP (a), lipoprotein (a).

### 3.5 Correlation analysis of serum ASGR2 levels

**Figure 3:**
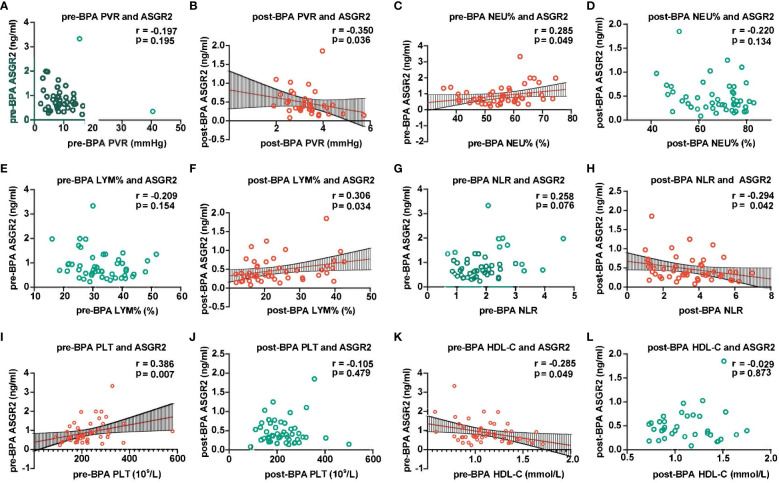
Association between ASGR2 and clinical parameters. ASGR2, asialoglycoprotein receptor 2; HDL-C, high-density lipoprotein cholesterol; PVR, pulmonary vascular resistance; LYM%, lymphocyte percentage; NLR, neutrophil-to-lymphocyte ratio; BPA, balloon pulmonary angioplasty; NEU%, neutrophil percentage. **(A)** pre-BPA PVR and ASGR2. **(B)** post-BPA PVR and ASGR2. **(C)** pre-BPA NEU% and ASGR2. **(D)** post-BPA NEU% and ASGR2. **(E)** pre-BPA LYM% and ASGR2. **(F)** post-BPA LYM% and ASGR2 **(G)** pre-BPA NLR and ASGR2. **(H)** post-BPA NLR and ASGR2. **(I)** pre-BPA PLT and ASGR2. **(J)** post-BPA PLT and ASGR2. **(K)** pre-BPA HDL-C and ASGR2. **(L)** post-BPA HDL-C and ASGR2.

According to the Spearman correlation analysis, pre-BPA ASGR2 levels were positively correlated with pre-BPA NEU% and PLT count but negatively correlated with HDL-C (R = 0.285, R = 0.386, R = -0.285, all *P* < 0.05). Post-BPA ASGR2 levels were positively correlated with post-BPA LYM % and the NLR (R = 0.306; R = -0.294, both *P* < 0.05, respectively). A significant positive Spearman correlation was observed between post-BPA ASGR2 levels and post-BPA PVR (R = -0.35, *P* < 0.05) (**Figure 3**).

### 3.6 Multiple linear stepwise regression analysis of ASGR2

For the pre-BPA treatment data, the equation ln(ASGR2*_pre-BPA_*) = -1.433 HDL-C *_pre-BPA_* + 1.013 PLT *_pre-BPA_* (R^2^ = 0.407, F = 15.812, *P* < 0.001) was obtained. Notably, NEU% was excluded from the analysis due to multicollinearity. Subsequently, for the pre-BPA treatment data, ln(ASGR2 *_post-BPA_*) = -2.879 + 0.388 ln(LYM% *_post-BPA_*) (R^2^ = 0.15, F = 6.016, *P* < 0.05) (**Table 4**).

**Table 4:** Stepwise linear regression of ASGR2.

Variable	*P*	Tol	R^2^	Adjusted R^2^	F
Pre-BPA (n = 48)					
PLT, ×10^9^/L	0.001		0.407	0.382	15.812
HDL-C, mmol/L	< 0.001				
NEU%, %	0.551	0.076			
Post-BPA (n = 48)					
Constant	< 0.001		0.150	0.125	6.016
LYM%, %	0.019				
NLR	0.185	0.042			
PVR, Wood U	0.063	0.969			

Tol, collinearity tolerance; ASGR2, asialoglycoprotein receptor 2; HDL-C, high density lipoprotein cholesterol; PLT, platelet; NEU%, neutrophil percentage; LYM%, lymphocyte percentage; NLR, neutrophil to lymphocyte ratio; PVR, pulmonary vascular resistance.

## 4 Discussion

This study aimed to utilize plasma proteomics to explore the molecular changes, pathway alterations, and potential novel biochemical markers associated with BPA in CTEPH patients. The findings of the study can be summarized as follows: (I) After successful BPA procedures, the serum levels of ASGR2, which were initially greater in CTEPH patients than in healthy individuals, significantly decreased. (II) Prior to BPA treatment, a correlation was observed between serum ASGR2 levels and PLT count as well as HDL-C levels. Following BPA treatment, serum ASGR2 levels were associated with LYM%. (III) Serum ASGR2 levels may be associated with PVR.

As a subunit of the asialoglycoprotein receptor (ASGPR), ASGR2 plays a significant role in cellular processes. Known as the Ashwell–Morell receptor, ASGPR is a transmembrane protein primarily expressed in hepatocytes that specifically recognizes N-acetylgalactosamine and galactose. Its main function is to internalize and degrade glycoproteins through desialylation, contributing to the maintenance of serum glycoprotein homeostasis (14). A strong correlation of ASGR2 with gastrointestinal tumors, including hepatocellular carcinoma, gastric cancer, and colorectal cancer has been reported (14). Moreover, emerging evidence suggests a potential association between ASGR2 and neuropsychiatric/neurodegenerative diseases, as well as hemophilia. Currently, there is no available research that establishes a connection between ASGR2 and PAH.

### 4.1 Relationship between ASGR2 and PLT count

Our study revealed a correlation between serum ASGR2 levels and PLT count in CTEPH patients before BPA intervention. Decreased PLT count and function have been recognized as factors that promote the development of CTEPH (15). In patients with CTEPH, PLTs demonstrate heightened activation, yet there is an intriguing phenomenon of reduced PLT aggregation and increased depolymerization. The activation of PLTs affects the production of proinflammatory chemokines and the aggregation of pulmonary interstitial macrophages, thereby contributing to the inflammatory state. The aggregation of PLTs and granulocytes in the peripheral blood further contributes to immune inflammation and the pathophysiology of CTEPH (16). The literature on the direct impact of ASGR2 in CTEPH patients is limited. However, studies suggest that the ASGR2 genotype may modulate von Willebrand factor, influencing the molecular link between inflammatory pathways and PLT adhesion during thrombus formation in CTEPH patients. However, further research is needed to fully understand the role of ASGR2 in CTEPH pathogenesis (17).

Our study revealed a correlation between the PLT count and ASGR2 level before BPA treatment. However, this correlation was no longer present after successful BPA treatment. These findings suggest that the initial abnormalities in PLT count and function observed in CTEPH patients are resolved by BPA intervention treatment, resulting in reduced inflammation and decreased PLT aggregation. The ASGR2 level may reflect the immune and inflammatory status of patients with CTEPH.

### 4.2 Relationship between ASGR2 and HDL-C

We observed a correlation between serum ASGR2 levels and HDL-C levels in CTEPH patients prior to BPA treatment. In CTEPH patients, dysfunctional HDL-C is associated with RV structure, PVR and proinflammatory effects (18–20). HDL-C levels are associated with peripheral blood leukocytes, including neutrophils, lymphocytes, and monocytes (21). The monocyte-to-HDL ratio is a novel marker of systemic inflammation in PH patients (21). The current understanding of the impact of ASGR2 on HDL-C formation and conversion, as well as its role in lipid metabolism and lipid levels, is still uncertain.

In our study, no significant change in HDL-C levels was observed pre- or post-BPA intervention. The correlation between ASGR2 and HDL-C, which was initially present prior to BPA treatment, disappeared after BPA treatment. Inflammation has been shown to reduce the levels of ApoA-I, the protein component of HDL-C, and impair the function of HDL-C, resulting in proinflammatory effects. Notably, ApoA-I has been found to inhibit IL-6 secretion by macrophages and attenuate IL-6-induced proliferation and migration of pulmonary artery endothelial cells (22). Macrophages at inflamed sites can express ApoA-I, potentially exerting anti-inflammatory effects without affecting serum HDL-C levels (19, 23).

Our study revealed that ApoA-I levels increased after BPA treatment in CTEPH patients, indicating a potential link between inflammation and HDL-C function. Prior to BPA, inflammatory processes in CTEPH patients may lead to decreased ApoA-I levels and dysfunctional HDL-C, contributing to vascular pathologies. Following BPA intervention, the elevated ApoA-I levels, together with macrophages, suppressed inflammation and restored normal HDL function without affecting HDL-C levels. These findings support the hypothesis that ASGR2 may serve as a marker reflecting the inflammatory status and hemodynamic levels in CTEPH patients through its association with HDL-C.

### 4.3 Relationships between ASGR2, LYM, NEU and the NLR

In our study, a significant correlation between ASGR2 expression and the percentage of NEUs was detected before BPA treatment, but this correlation disappeared after treatment. Neutrophils and their products, including myeloperoxidase, proteases, and neutrophil extracellular traps (NETs), are key contributors to PH. They degrade vascular elastin, drive vascular remodeling, amplify the leukocyte response, and modify the local inflammatory environment (24). Additionally, neutrophils can impair the antioxidant and anti-inflammatory functions of HDL-C, potentially leading to the development of atherosclerosis (25). The interaction between NETs and platelets during programmed cell death promotes thrombosis formation in PAH patients. In addition, neutrophils can also impair the antioxidant and anti-inflammatory functions of HDL-C, leading to the development of atherosclerosis (26).

In our study, no significant correlation was found between ASGR2 expression and the LYM% or NLR before BPA treatment. However, after BPA treatment, a correlation between ASGR2 expression, LYM%, and the NLR was observed. Immune system dysfunction plays a crucial role in the pathogenesis of PH, as evidenced by alterations in circulating T-cell subsets. T lymphocytes infiltrate the pulmonary arteries of CTEPH patients and secrete cytokines, leading to damage to newly formed blood vessels and exacerbating disease progression. The upregulation of T lymphocytes and cytokines promotes the recruitment of inflammatory cells and contributes to the proliferation of smooth muscle and endothelial cells (27–29). Peripheral blood cells infiltrate affected lung tissues, leading to inflammatory cell infiltration and impacting peripheral blood cell counts and ratios (30). The decrease in the percentage of peripheral blood LYMs following BPA intervention indicated a successful improvement in immune signaling, leading to reduced lymphocyte activation and proliferation. Furthermore, importantly, the NLR is a reliable biomarker for the diagnosis of PH, risk stratification, and prognosis prediction (31). Therefore, ASGR2 may be an indicator of immune function and inflammatory status in CTEPH patients, as reflected by its association with LYM, NEU, PLT, HDL-C and the NLR.

Multiple linear stepwise regression analysis revealed no significant relationships between ASGR2 and the NEU% or NLR, likely due to multicollinearity. The LYM% explained 15% of the variation in ASGR2 after BPA treatment, while the combination of PLT and HDL-C explained 40.7% of the variation before BPA treatment. Nevertheless, there are still unidentified factors that influence ASGR2, highlighting the need for further research.

### 4.4 Relationship between ASGR2 and PVR

In our study, the serum levels of ASGR2 may be associated with PVR. The PVR is associated with the NLR, LYM, and pulmonary vascular remodeling (32, 33). Furthermore, PVR is negatively correlated with the levels of IL-7, a cytokine necessary for B-cell maturation and regulatory T-cell survival (34). Taken together, these findings suggest that PVR levels may be influenced by lymphocyte immune function and inflammation. We observed that the *P* value for PVR was 0.063, indicating a trend toward significance. The exclusion of PVR from the multiple regression analysis was primarily due to the limited sample size, which may have impacted the statistical power to detect a significant relationship.

### 4.5 Limitations

This study may be limited by its small sample size. Although our study revealed statistically significant correlations between ASGR2 and NEU, LYM%, and the NLR, the strength of these associations was modest. This may be attributed to the limited sample size employed in our study. To enhance the robustness and generalizability of our findings, future research should focus on recruiting a larger sample size for further validation and replication of our results.

## 5 Conclusions

The serum ASGR2 concentration may be a biomarker for BPA treatment effectiveness in CTEPH patients. The pre-BPA serum level of ASGR2 in CTEPH patients was associated with HDL-C and the PLT count. The post-BPA serum level of ASGR2 was correlated with the LYM%, which may reflect aspects of immunity and inflammatory status.

## Data availability statement

The original contributions presented in the study are publicly available. This data can be found here: https://ngdc.cncb.ac.cn/omix/release/OMIX005982.

## Ethics statement

The studies involving humans were approved by the ethics committee of the Shanghai Pulmonary Hospital. The studies were conducted in accordance with the local legislation and institutional requirements. The participants provided their written informed consent to participate in this study.

## Author contributions

W-JX: Formal Analysis, Writing – original draft, Methodology. SW: Formal Analysis, Writing – original draft, Validation. Q-HZ: Validation, Writing – original draft, Formal Analysis. J-YX: Writing – original draft, Methodology. X-YH: Writing – original draft. S-GG: Writing – original draft, Resources. JH: Writing – original draft. H-LQ: Writing – original draft. C-JL: Writing – original draft. JX: Writing – original draft. H-TL: Writing – original draft. Z-PL: Writing – review & editing. LW: Writing – original draft, Resources. YS: Writing – review & editing. Y-LZ: Writing – review & editing, Methodology. RJ: Conceptualization, Funding acquisition, Writing – review & editing.
